# The Scientific Aspects of the Medical Curriculum

**Published:** 1909-09-04

**Authors:** 


### THE SCIENTIFIC ASPECTS OF THE MEDICAL CURRICULUM.

A famous French physicist has said that each generation laughs at the generalisations of the preceding one. If this hold good in the lofty domain of pure science, there is every excuse for frequent rectification of tradition in medicine, and therefore in the preparation for a medical career. Medicine has been loosely described as applied science. This description, impossible fifty years ago, and even now distinctly meagre, will never come to be a definition. Still, there are certainly many truths in biology, physics, botany, and physiology which the student of medicine must grasp firmly in order to obtain a foundation for his later and more strictly professional knowledge—a consequence, or rather, one of the many consequences, of the march of science during the latter decades of the nineteenth century of which the general educated public is not yet aware. The prevalent idea as to medical students' studies is still the old "walking the hospitals"; and many people are surprised if, after two years at a medical school, a friend or relative has not some little practical knowledge of his art—cannot, for instance, tell them what is good for a gumboil. They think, to put it colloquially, that in two years he should surely have got as far as gumboils; whereas the attention of the unfortunate individual in question may have been devoted pretty exclusively during the whole of that time to such things as test-tubes and the internal arrangements of the earthworm. The foundation of modern physiology by Johannes Müller, with Schwann's enunciation of the cell theory, and Virchow's revelation of its bearing on the processes of disease, soon made scientific training and study necessary preliminaries to the learning of rational therapeutics. Then Pasteur discovered the microbe, and, in their various departments and degrees, Koch, Ehrlich, Laveran, and (to be patriotic for once) Lister and Wright, established by scientific experiment new views on every point in medicine. To repeat some of the simpler and most practically significant of these experiments for himself, much as a schoolboy may those of Faraday, is part of every medical student's curriculum nowadays; and hence the purely clinical period—the period for the examination of gumboils—tends to be still further deferred.

Current prospectuses of the various schools of medicine throughout the country accordingly show the subjects of medicine and surgery proper to come rather late on the schedule of instruction, and moreover to comprise courses in morbid anatomy and bacteriology. When taking out these courses the student resumes in a great measure his earlier line of work, work in which the microscope plays a large part and to a less extent also the apparatus of the chemical laboratory. Natural aptitude for the use of these instruments consists in the possession of what is familiarly known as "technique," *i.e.* neat-handedness and orderly method in small mechanical procedures, together with patience and tenacity. The boy who is fond of photography will find himself at home in a laboratory; but "technique," although of the greatest use to a medical student, does not always go along with high mental capacity. Quite clearly it is to some extent within the power of every shop assistant who can do up a parcel neatly, while, if the photographer's fingers are to be valued against the philosopher's brain, there is no doubt as to what the verdict will be. But medical microscopy is generally described as interesting to the average person, and a relief from the rather heavy reading necessary in latter-day medicine, where facts have outgrown the scope of oral instruction, and must perforce be sought for in text-books which increase steadily in bulk every two or three years.

To obtain desired laboratory reactions with constancy, too, is a small test of honesty, practical ability, and perseverance—all qualities needful to the good clinician. A parent should, however, never look upon a brilliant academic career in scientific medicine as a guarantee of his son's future scientific eminence, although it is certainly of much immediate advantage. Originality and imagination, the essential attributes of the real investigator, are different matters altogether. As a rule, great advances are achieved by simple methods, their prevision being the sole indispensable antecedent. Lord Kelvin's class demonstrations generally went wrong; in fact, he was far from being a good practical experimentalist. Faraday "divined the truth" with inferior apparatus. Koch's method of identification of the tubercle bacillus was by no means a complicated process; before him, Ehrlich for one had made the note to see what was the true nature of the little red rods in the microscope field. Nevertheless, in this country at any rate, small allowance is made, in any form of instruction, for originality, and the man well thought of is the one who has come successfully through the academic mill.

Judged by the hard and almost fatal test of what of value in after life remains to the educatee, no doubt medical academic instruction, being highly technical, compares favourably enough with the other kinds. But still expertness, the result of laboratory training, is perhaps rated too highly, and the inspiring influence of a real scientific enthusiast upon kindred souls, though often mentioned, is yet under-valued. Unfortunately, too, it happens only too frequently that those whose qualifications, assessed by the examination test, stand highest, yet lack some essential element of the first-class clinical worker, without which no amount of book-learning is of any value. There can hardly be a medical school in the kingdom in which it is not possible to single out one surgeon at least whose academic distinctions stand as high as his surgical performances are the reverse; and whose position, gained as a young man, could be better filled by many of those whom, as students, he seemed easily to outdistance. The history of medical discovery makes it safe to predict that the problems of cancer or of leprosy will be solved, not by deputies and recorders, but by minds which, in Hazlitt's fine phrase, can "invent according to nature," and thereby command success upon their own conditions.

